# Safety and Efficacy of the New Combination Iron Chelation Regimens in Patients with Transfusion-Dependent Thalassemia and Severe Iron Overload

**DOI:** 10.3390/jcm11072010

**Published:** 2022-04-03

**Authors:** Raffaella Origa, Monia Cinus, Maria Paola Pilia, Barbara Gianesin, Antonietta Zappu, Valeria Orecchia, Maria Grazia Clemente, Carla Pitturru, Anna Rita Denotti, Francesco Corongiu, Simona Piras, Susanna Barella

**Affiliations:** 1 SSD Talassemia, Ospedale Pediatrico Microcitemico ‘Antonio Cao’, Via Jenner s.n., 09121 Cagliari, Italy; mariap.pilia@aob.it (M.P.P.); antonietta.zappu@aob.it (A.Z.); valeria.orecchia@aob.it (V.O.); maria.g.clemente@aob.it (M.G.C.); carla.pitturru@aob.it (C.P.); anna.r.denotti@aob.it (A.R.D.); francesco.corongiu@aob.it (F.C.); simona.piras@aob.it (S.P.); susanna.barella@aob.it (S.B.); 2 Dipartimento di Scienze Mediche e Sanità Pubblica, Università di Cagliari, Cittadella Universitaria di Monserrato-Blocco I, SS 554 Bivio Sestu, 09042 Monserrato, Italy; moniacinus@gmail.com; 3 Fondazione For Anemia, Via Garibaldi 7 C3, 16124 Genova, Italy; barbara.gianesin@galliera.it

**Keywords:** combined therapy, iron chelation, MRI, desferrioxamine, deferiprone, deferasirox, compliance

## Abstract

The aim of this study is the evaluation of the safety and the efficacy of long-term combination therapy deferasirox plus desferrioxamine and deferasirox plus deferiprone in a large group of transfusion-dependent thalassemia patients with high values of serum ferritin and/or magnetic resonance, indicative of severe liver and cardiac iron accumulation. Sixteen adults with transfusion-dependent thalassemia were treated simultaneously with deferasirox plus desferrioxamine, while another 42 patients (seven children) were treated with deferasirox plus deferiprone. The hepatic and cardiac iron overload was assessed prior to treatment and then annually with magnetic resonance imaging, and the serum ferritin was measured monthly. Adverse events were checked at each transfusion visit. The safety of both the combinations was consistent with established monotherapies. Both treatments were able to decrease the serum ferritin and liver iron concentration over time, depending on the level of compliance with therapy. Cardiac iron measured as R2* did not significantly change in patients treated with deferasirox plus desferrioxamine. Most patients with MRI indicative of myocardial siderosis at the beginning of treatment reached normal values of cardiac iron at the last determination if treated with deferasirox plus desferrioxamine. The greatest limitation of these therapies was low patient adherence to the two drugs, which is not surprising considering that the need for an intensive chelation is generally linked to previous issues of compliance.

## 1. Introduction

Thanks to the availability of three iron chelating drugs and the evidence that they can be used in combination when it is necessary to intensify chelation or make it more tolerable, iron chelation therapy can be personalized and tailored to the patient with thalassemia.

At least 10 different regimens for controlling iron overload have been identified [1].

The desferrioxamine (DFO) plus deferiprone (DFP) combination has been shown to be able to reduce myocardial iron accumulation, liver iron concentration (LIC), and serum ferritin with Level A evidence [1]. On the contrary, the impact of newer treatment schedules, especially for regimens involving a combination of deferasirox (DFX) plus DFO or plus DFP, remains an area of uncertainty.

The simultaneous use of DFX and DFO was reported for the first time in 2013 by Lal et al. [2], who safely and successfully treated 22 patients with transfusion-dependent thalassemia and persistent iron overload or organ damage. Moreover, the Hyperion trial showed the safety and efficacy of the combination therapy with DFX plus DFO followed by monotherapy with DFX in patients with severe transfusional cardiac siderosis [3]. Nevertheless, there are few other published studies on this combined treatment, which are heterogeneous [4,5,6,7,8]. However, in those in which the effect on heart and liver iron were evaluated, a tendency to decrease in hepatic iron may be noted. The results in terms of heart iron were more variable.

Paradoxically, the number of reports on the safety and efficacy of the combination DFX plus DFP is lower than the number of reports on the DFX plus DFO combination, although the simultaneous use of the two oral chelators is characterized by the greatest synergism in cellular models (about 60% of mobilized iron attributable to synergistic interaction) [9].

All but one of the reports on DFX and DFP association were from the Middle East or India, and involved a limited number of patients, especially of pediatric age [10,11,12,13,14,15,16]. Moreover, different drug dosages and endpoints make these studies hard to compare.

The present study aims at evaluating the safety and efficacy, with special attention to the effects on heart and liver iron, of long-term combination therapy DFX plus DFO and DFX plus DFP in a large group of transfusion-dependent patients. To our knowledge, this is one of the largest studies on the combined chelation therapy in terms of both number of patients and follow up duration in the clinical setting.

## 2. Materials and Methods

This was a retrospective cohort study utilizing Webthal^®^, a web-based clinical records software program developed to help physicians across Italy in daily clinical patient management. At each center, Ethics Committee approval was obtained and written informed consent for data collection and use were retrieved from the patients. Data on patients with beta thalassemia major and a documented simultaneous use of two iron chelators apart from the association DFP plus DFO were collected.

All of the patients were regularly transfused and iron chelated at Day Hospital Trasfusionale, SSD Talassemia-Ospedale Microcitemico “A.Cao”, Cagliari (Italy) according to internationally accepted guidelines [17].

The hepatic and cardiac iron overload were assessed prior to treatment, and then annually with T2* magnetic resonance imaging (MRI scanner Siemens—1.5 T, software used CMRTools). The liver iron concentration (LIC) was estimated by converting T2* measurements to mg/g dry weight (d.w.) with the formula: LIC = 0.202 + 25.4/liver-T2*. Ref. [18] R2*, the reciprocal of T2* was used to measure the cardiac iron variations over time.

Serum ferritin was measured monthly on venous samples using an automated immunoassay system (IMMULITE 2000).

Heart function was monitored once or twice a year (as prescribed by the cardiologist) via echocardiography, measuring the left ventricular ejection fraction (LVEF).

Adherence to and acceptance of the chelation therapy (compliance) was evaluated according to pharmacy records of the dispensed drug. It was classified as good if the mean number of the doses taken was ≥80% of those prescribed, average if it was ≥50 < 80%, and poor if it was <50%.

Adverse events were checked at each transfusion visit.

Descriptive statistics were performed with mean ± standard deviation and range (min–max), counts, and proportions. Repeated measured ANOVA was used to analyse the changes in the serum ferritin, LIC, and heart T2* from basal; multiple pairwise comparisons between groups (with Bonferroni correction) was used as a post-hoc test to identify which groups were different. The effect of time was also studied for each level of compliance. The percentage of reduction between the basal and last follow-up evaluation of ferritin and LIC with respect to the basal were calculated for each patient, and their dependence to other covariates were investigated with univariate and subsequently multivariate regression analysis. A type 1 error (α) of 0.05 was considered to assess statistical significance.

## 3. Results

### 3.1. Combination Therapy DFX plus DFO

From 2018, 16 adult patients aged 38 ± 6 years (range 29–50) were prescribed combined iron chelation treatment with DFO and DFX, 11 of which were female.

DFX plus DFO combination was considered in case of serum ferritin and/or LIC and/or myocardial T2* indicative of severe iron accumulation; suboptimal chelation with monotherapies; and rejection, intolerance to, or severe side effects with DFP.

In more detail, the main rationale for choosing DFX plus DFO therapy was a previous suboptimal chelation associated with a history of agranulocytosis, neutropenia, or gastric intolerance to DFP in 12 patients. In the other four cases, the DFX plus DFO chelation regimen was chosen in patients with no history of major side effects or serious intolerances to DFP based on the physician’s preference (good results in other patients in the treatment of liver overload) in agreement with the patient.

Before the start of this therapy, all but one patient on DFP plus DFO were on monotherapy (DFO in 2 patients, DFP in 2 patients, and DFX in 11 patients).

The mean DFX starting dose was 23 ± 7 mg/kg per day. DFO was given subcutaneously in all but one patient (intravenous administration via port-a-cath) at 42 ± 8 mg/kg per day over 10–24 h/day, 2 to 7 days per week according to the ferritin level and the organ iron overload over time.

The duration of treatment varied from 239 days to 2305 days, with a total duration of 16,090 days, equal to 44.1 patient-years. One patient was treated for less than 1 year, four had between 1–2 years of treatment, another four had between 2–3 years of treatment, and seven had more than 3 years. At the time of the analysis, combination treatment was still ongoing in 12 patients, while it had been interrupted in four (poor results and compliance in two, patient decision in one, and port-a-cath infection with intolerance to subcutaneous administration in one).

Only one patient did not experience adverse events related to chelation; six had gastrointestinal discomfort in association with DFX; three had at least one creatinine value above the upper normal value for sex, age, and weight; four had repeated urinary protein/creatinine ratio >0.5; and four local side effects from DFO.

Adherence to combination therapy was good for both chelators in three patients, average in six patients (good for DFX and average for DFO in four and average for both in two), and poor in seven.

The mean blood consumption, which was 147 ± 49 mL/kg/year of pure red cells before the start of DFX plus DFO, did not significantly change during the treatment (143 ± 36 mL/kg/year of pure red cells, *p* = 0.6).

We observed a significant reduction of serum ferritin after 12 months and 36 months of therapy. On the contrary, the reduction of LIC and cardiac R2* did not reach the level of statistical significance. However, the same analysis performed for each level of compliance showed a significant decrease in serum ferritin and liver iron over time limited to the patients with average/good adherence to therapy (*p* = 0.04 and *p* = 0.03, respectively).

Cardiac iron was present in 10/16 patients at the beginning of treatment and in 8/15 at the last T2* determination (*p* = 0.88), and its variation over time was not significant, irrespective of compliance.

During DFX plus DFO treatment, LVEF, which was normal (≥56%) in all patients, remained stable (from 67 ± 6 to 68 ± 5%, *p* = 0.4).

**Figure 1:**
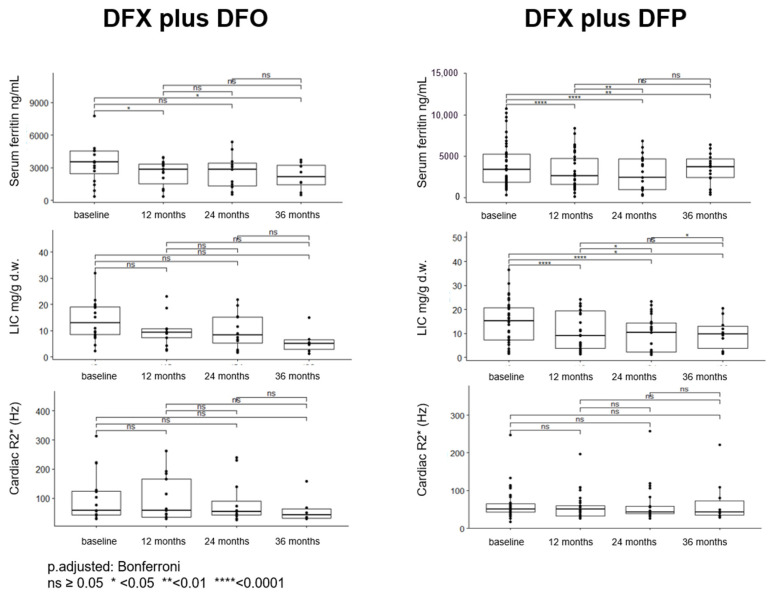
Overall efficacy data for combination iron chelation treatments of deferasirox (DFX) plus desferrioxamine (DFO) and DFX plus deferiprone (DFP). Data are displayed as point distribution and box plots reporting median and quartiles.

Comprehensive efficacy data on the association DFX plus DFO are shown in Table 1 and Figure 1.

### 3.2. Combination Therapy DFX plus DFP

Here, 42 patients, 35 adults (37 ± 6 years, range 26–54) and 7 children (9 ± 3 years, range 4–13, 2 < 6 years; 3 ≥ 6 < 12 years; and 1 ≥ 12 < 18 years), were prescribed the combined therapy of DFX plus DFP from 2014. Before the start of the combination treatment, 9 patients were on DFP, 14 on DFX, 1 on alternating DFO and DFP, 6 on alternating DFX plus DFP, 6 on combination DFP plus DFO, and 2 on DFX plus DFO. All patients had a significant iron overload and refused DFO, or had local or systemic intolerance to it. Both drugs were prescribed daily, with DFP at a mean dose of 93 ± 13 mg/kg divided in three administrations and DFX film coated tablets at a mean dose of 23 ± 5 mg/kg once a day.

The total duration of therapy was 34,953 days, equal to 95.8 patient-years, varying from 56 to 1786 days in single patients. Ten patients were treated for less than one year, seven had between 1–2 years of treatment, ten had between 2–3 years, eleven had between 3–4 years, and four more than 4 years.

Adherence to chelation therapy was good in 21 patients, average in 14 (in eight cases due to DFP, in three due to DFX, while in three cases compliance was between 50% and 80% for both the oral chelators), and poor in 7 (in four cases due to DFP, in one due to DFX, while in three cases compliance was <50% for both drugs).

Seven patients had stopped combination therapy at the time of the analysis. Reasons for interruption were worsening of iron overload (poor compliance) in one patient who was switched to combined therapy DFP plus DFO, and improvement of iron parameters with switch to monotherapy in five. Two patients interrupted the combination therapy DFX plus DFP because of adverse events (repeated neutropenia linked to DFP in one case and nephrotic proteinuria associated with amyloidosis in another).

Six patients experienced gastrointestinal discomfort; three had at least one creatinin value above the upper normal value for sex, age, and weight; and four had repeated urinary protein/creatinine ratio >0.5. Two patients encountered repeated episodes of neutropenia.

The mean blood consumption, which was 159 ± 44 mL/kg/year of pure red cells before the start of DFX plus DFP, did not significantly change during the treatment (159 ± 40 mL/kg/year of pure red cells, *p* = 0.9).

Serum ferritin decreased after 12 months (*p* < 0.0001), and the significant reduction was maintained after 24 and 36 months. Serum ferritin decreased only when compliance was average/good (35 subjects, *p* = 0.0002), and did not in the seven with poor adherence to therapy.

LIC showed a trend similar to that of serum ferritin—we observed a significant decrease over time, considering the whole group and the patients with good compliance (*p* = 0.006) but not those with poor compliance (0.7).

The cardiac R2* of the whole population did not change significantly during oral combined treatment. However, the patients with cardiac iron (heart T2* < 20 ms), who were 20 out of 38 at the start of the DFX plus DFP treatment, became 7 out of 30 at the last determination (*p* = 0.028).

All 16 patients who had good compliance with treatment (one with severe cardiac iron overload, seven with mild/moderate heart overload, and eight with no myocardial iron) and repeated MRI over time, showed normal levels of cardiac iron at the last evaluation. LVEF, which was normal (≥56%) in all but one patient, did not significantly change (from 64 ± 7 to 68 ± 6%, *p* = 0.2).

Comprehensive efficacy data on the association DFX plus DFP are shown in Table 1 and Figure 1.

The multivariate analysis highlighted and confirmed that adherence to therapy (average/good vs. poor) shapes the percentage reduction of serum ferritin in both the combination therapies (*p* = 0.04), as well as the percentage reduction of LIC (*p* = 0.011). The other variable conditioning the percentage reduction of serum ferritin is its value at baseline (higher the serum ferritin value at baseline, higher its percentage reduction, *p* = 0.001). More detailed results of the univariate and multivariate analysis are reported in Table 2.

## 4. Discussion

Nowadays, most thalassemia patients can reach the goal of a normal or near-normal level of serum ferritin and normal concentrations of cardiac and hepatic iron thanks to the possibility of directly measuring the accumulation of iron in the organs and the availability of three iron chelators, two of which can be administered orally. Furthermore, the possibility of an intensive DFP plus DFO chelation has permitted a relatively rapid reduction of iron overload and has increased the survival rate of patients [19].

However, problems with adherence to chelation therapy or side effects to one or more of the chelating drugs make it difficult to achieve the desired goal in a subgroup of subjects, with the risk of disabling complications and a reduced rate of survival.

In an attempt to resolve these issues, new chelation regimes have been tested over time by associating the chelators alternately over the course of the week or by combining two drugs other than the more well-known DFO and DFP on the same day.

Ten different regimens for controlling iron overloading in transfusion-dependent thalassemia have been identified, thus allowing individualization of the chelation therapy for each patient, tailored according to their unique characteristics and needs [1].

Nevertheless, a significant grey area remains in the chelation regimens introduced in clinical practice more recently, and, with particular reference to new combined therapies, the published data usually refer to small patient series, and the drug dosages, schedules, and considered outcomes differ.

In 2013, Grady et al. demonstrated that the daily use of DFX with 2–3 days of DFO treatment placed patients into a net negative iron balance [20]. Albeit heterogeneous, almost all subsequent analyses have confirmed the efficacy of the combination of DFX plus DFO in reducing serum ferritin and removing hepatic iron [2,3,4,5,6,7,8]. An exemption is the randomized study by Moravi et al. (2014), in which the serum ferritin reduction was not significantly greater in patients on combination therapy (DFO at the dosage of 50 mg/kg 3 times a week plus oral DFX 20–40 mg/kg per day) than in those on DFO monotherapy [21].

Most studies, although involving a small number of patients in some cases, have also confirmed a positive effect on cardiac iron. In HYPERION, an open-label single-arm prospective phase 2 study, the combination DFX plus DFO, followed by optional switch to DFX monotherapy when achieving heart T2* > 10 ms, was evaluated in 60 patients with myocardial T2* 5- < 10 ms and LVEF ≥ 56% at baseline. Heart T2* increased from 7.2 ms at baseline to 7.7 ms at 12 and 9.5 ms at 24 months. Patients (17 of 60; 28.3%) achieved mT2* ≥ 10 ms and ≥ 10% increase from baseline at month 24. Moreover, LIC decreased substantially from a baseline of 33.4 to 12.8 mg Fe/g dry weight at month 24 (−52%) and LVEF remained stable [3].

In another clinical trial, Lal et al. [2] showed a median LIC decrease by 31% and a median ferritin decrease by 24%, as well as a significant improvement in MRI T2* in 18 subjects completing 12 months of DFX plus DFO therapy. Similarly, a marked reduction in LIC (from 11.44 to 6.54 mg/g d.w.) and serum ferritin (from 2254 to 1346 ng/mL) was reported by Cassinerio et al. [4]. As in the above-mentioned studies, an improvement in cardiac T2* values was detected (from 19.85 ± 12.46 to 26.34 ± 15.85 ms).

Unlike Lal et al. [2] who reported an average compliance with prescribed therapy (monitored by transfusions visit interviews) of 89% for DFO and 94% for DFX, Arandi et al. [5] who reported a treatment adherence rate of 100% and Eghbali et al. [6] who defined the compliance of their patients on DFX plus DFO as “acceptable”, our analysis highlights that the greatest limitation of this therapy is patient adherence to the two drugs, and especially to DFO, which is not surprising considering that the need for intensive chelation is generally linked to previous compliance issues.

The safety of the combination DFX plus DFO was consistent with established monotherapies and no distinctive warnings have emerged, confirming what has been reported in the other studies. However, the high percentage of patients with abdominal discomfort due to DFX and with local side effects at the DFO infusion site may contribute to explain the poor adherence to the two drugs.

Although the number of patients on DFX plus DFO is too low to draw definitive conclusions, the possibility of a reduction in terms of ferritin and LIC appears to be precisely influenced by compliance. However, the non-significant increase of cardiac T2* regardless of compliance is consistent with the lack of patients who normalized their myocardial T2* over time, suggesting that adhering to this type of therapy for an extended period of time may be particularly challenging, as well as to a peculiar slowness of this combination regarding the clearance of cardiac iron. Nevertheless, LVEF remained normal in all patients and only one young woman with severe heart iron overload and poor compliance with therapy experienced a first instance of arrhythmia, confirming previous works [3].

Similar to other scholars in previous experiences, we have also the DFX plus DFP combination therapy to patients with a heavily iron load. A recent pharmacokinetic study showed that one oral dose of DFX (30 mg/kg) along with two doses of DFP (30 mg/kg/dose) on the same day could increase the total iron excretion to a higher level than that resulting from monotherapy [20]. This combination may increase DFX plasma levels as previously mentioned by Vlachodimitropoulou Koumoutsea et al., and DFP may have an addictive effect on iron removal with a suggested shuttle effect similar to that assumed for the combination DFP plus DFO [9,20]. Moreover, from a theoretical point of view, the fact that both chelators can be administered orally should improve patient adherence to therapy.

However, no significant changes in LIC and heart T2* were reported by Karami et al. (2017) and Divakar et al. (2021), probably due to an inadequate sample size [14,16]. Among the six patients treated for 12 to 18 months by Hammond et al., three showed an improvement in T2* and two in liver iron [15]. In the same report, the authors claimed that the efficacy of combination oral chelation may be limited by adverse effects and challenges to adherence.

In our experience, 52% of patients treated with the two oral drugs had good compliance and 17% had poor compliance in comparison with the 19% and 44%, respectively, of patients on combination treatment with DFO and DFX. On the one hand, this is the confirmation that, independently from its tolerability and convenience, any combined chelation therapy is difficult for patients to comply with and should be considered only after monotherapies have failed, as well as in association with extensive support and continuous education.

On the other hand, the results obtained in our patients, when considered as a whole and divided according to adherence to therapy, are indicative of the fact that the DFX plus DFP therapy is also effective in terms of serum ferritin and LIC reduction when the compliance to treatment is good or at least intermediate.

On the contrary, the response of combination therapy with the two oral iron chelators in terms of cardiac iron reduction remains questionable: considering the R2* change over time, regardless of whether cardiac iron is present, it does not seem to vary in a significant manner. However, most patients with an MRI indicative of myocardial siderosis at the beginning of treatment reached normal values of cardiac iron at the last evaluation, especially if the adherence to treatment was average or good.

In this regard, the results of our study compared with those already published on combination therapy DFP plus DFO [22,23,24] do not seem to confirm what was reported by Elalfy et al., who reported that both DFX plus DFP and DFP plus DFO combinations were equally effective at reducing serum ferritin and LIC in 96 young thalassemia major patients with severe iron overload on a prospective randomized trial, while DFX plus DFP was proven to be superior in improving cardiac T2* [25]. As possible explanations, we would like to emphasize the different age of patients (10–18 years in the randomized trial and mostly adults in our study) and the gap between formal clinical trials and clinical setting. Supporting this, although greater in those treated with the two oral chelators, treatment compliance was very good in both groups of patients treated by Elalfy et al. (95% vs. 80%, respectively, *p* < 0.001), much better than in our patients.

Ultimately, the characteristics of patients in which these types of therapies are proposed in real life make it difficult to discriminate how much of the response to therapy is conditioned by the intrinsic properties of the two associated drugs, and how much it is related to compliance and by complete adherence to the therapeutic proposal. Patients are often not compliant to the proposed combined chelation regimens in the same way that they were not to previous monotherapies.

**Figure 2:**
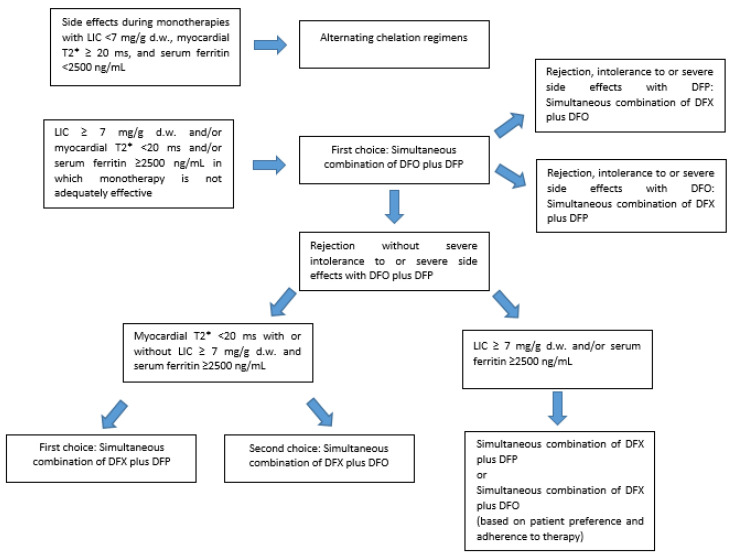
Proposed treatment algorithm for patients with transfusion-dependent thalassemia with persistent or increasing iron overload in which monotherapy is not adequately effective. DFX: deferasirox; DFO: desferrioxamine; DFP: deferiprone; LIC: liver iron concentration.

While alternating iron chelation regimens may be taken into consideration in case of unmanageable side effects from monotherapies, combined iron chelation treatments may be valid options in patients with persistent iron overload, when an intensification of iron chelation treatment is mandatory (Figure 2). However, the available evidence, including from this work, does not allow for a conclusion for the non-inferiority of the simultaneous DFX plus DFP and DFX plus DFO therapies vs. the DFP plus DFO association.

When myocardial iron overload is detected via MRI, the simultaneous use of DFO and DFP is the treatment of choice. If the patient refuses DFO, the association DFX plus DFP may lead to a reduction of cardiac iron, if the patient adheres to the proposed therapy over time without any drop in compliance. In the case of myocardial iron, especially if severe, the combination DFX plus DFO should be proposed as the last choice.

Both the newer combined iron chelation treatments may be considered as valid options in patients with high serum ferritin values and severe or moderate liver iron overload. The decision to start a DFX plus DFP or a DFX plus DFO combined therapy in the case of elevated ferritin values or LIC in the absence of severe cardiac iron should be evaluated case by case, based also on previous side effects and intolerance to monotherapies and the patient’s willingness to actually carry out the proposed therapy. A collaborative doctor–patient relationship able to improve patient empowerment might increase patient adherence, as well as the likelihood that the new combination therapies will be effective.

## 5. Conclusions

Both the newer combined iron chelation treatments can be considered as options in patients with iron overload not responsive to monotherapies. Neither therapy appears to be associated with significant safety and tolerability issues. For both therapies, the effect on hepatic iron appears to be more rapid and more pronounced than the effect on cardiac iron, as long as compliance with therapy is at least average. The available data are insufficient to confirm that the combination of the two oral chelators may result in effects on cardiac iron comparable to those of the combination of DFP plus DFO.

Larger multicenter studies are needed to shed further light on the advantages and limitations of the simultaneous use of DFO and DFX and of the two oral chelators.

## Figures and Tables

**Table 1:** Mean ± SD of serum ferritin, LIC, and cardiac R2* at different times for patients treated with DFX plus DFO and DFX plus DFP.

		Patients (*n*°)	DFX plus DFO	Patients (*n*°)	DFX plus DFP
Serum ferritin ng/mL	Baseline	16	3472 ± 1820	42	4031 ± 2696
	12 months	15	2455 ± 1240	32	3256 ± 2235
	24 months	11	2594 ± 1591	25	2821 ± 2062
	36 months	7	2190 ± 1239	11	3440 ± 1860
LIC mg/g d.w.	Baseline	16	13.8 ± 7.65	32	14.6 ± 8.75
	12 months	15	10.3 ± 6.30	29	10.7 ± 7.80
	24 months	11	9.93 ± 6.85	23	9.45 ± 7.58
	36 months	7	5.9 ± 4.91	10	9.73 ± 6.61
Cardiac R2* (Hz)	Baseline	16	98.6 ± 84.5	32	62.5 ± 40.0
	12 months	15	97.4 ± 78.5	29	57.2 ± 35.0
	24 months	11	85.7 ± 75.5	23	62.7 ± 49.9
	36 months	7	61.4 ± 49.3	10	67.9 ± 59.4

DFX: deferasirox; DFO: desferrioxamine; DFP: deferiprone; LIC: liver iron concentration.

**Table 2:** Univariate and multivariate analysis of the association between type and duration of therapy, demographic factors, iron status, and compliance with the percentage reduction of serum ferritin (a) and LIC (b).

**Percentage Reduction of Serum Ferritin (a)**	**Univariate Model**		**Multivariate Model**	
Variable	Β-coefficients (95% CI)	*p*-Value	Β-coefficients (SE)	*p*-Value
Type of therapy (DFX + DFP vs. DFX + DFO)	7.2 (−14.5; 28.8)	0.51		
Compliance (low vs. medium/good)	−19.9 (−42; 2.2)	0.077	−21.04 (−41; −0.8)	0.04180
Duration of therapy	0.50 (−0.07; 1.1)	0.085		
Age at the start of therapy	−0.027 (−0.89; 0.84)	0.95		
Ferritin at basal	0.0060 (0.0023; 0.0096)	0.0017	0.0061 (0.0025–0.0096)	0.00105
Iron input during combination therapy	0.28 (0.006; 0.56)	0.045		
**Percentage Reduction of LIC (b)**	**Univariate Model**		**Multivariate Model #**	
Variable	β-coefficients (95% CI)	*p*-Value	β-coefficients (SE)	*p*-Value
Type of therapy (DFX + DFP vs. DFX + DFO)	25.8 (4.6; 47)	0.018	12.9 (−7.3; 63.2)	0.20
Compliance (LOW vs. MEDIUM/GOOD)	−41.4 (−62.1; −20.7)	0.00022	−37.3 (−58.8; 33.2)	0.0011
Duration of therapy	0.15 (−0.58; 0.88)	0.67		
Age at the start of therapy	−0.25 (−1.28; 0.78)	0.63		
LIC at basal	0.75 (−0.49; 1.99)	0.23	0.74 (−0.32; 1.8)	0.17
Iron input during combination therapy	0.17 (−0.12; 0.45)	0.24		

95% CI: 95% confidence interval; DFX: deferasirox; DFO: desferrioxamine; DFP: deferiprone; LIC: liver iron concentration. # The multivariate model was selected with the initial inclusion of covariates with *p*-value > 0.2 at the univariate also including the covariates “LIC at basal” and “consumption of iron during the therapy”, and subsequently removing the variables not significative at the multivariate.

## Data Availability

The data presented in this study are available upon request from the corresponding author.
